# A Resonant Coupler for Subcutaneous Implant †

**DOI:** 10.3390/s21238141

**Published:** 2021-12-06

**Authors:** Sen Bing, Khengdauliu Chawang, J.-C. Chiao

**Affiliations:** Electrical and Computer Engineering, Southern Methodist University, Dallas, TX 75205, USA; sbing@smu.edu (S.B.); kchawang@mail.smu.edu (K.C.)

**Keywords:** resonator, tuning, implant, subcutaneous, wireless power transfer, implant localization

## Abstract

A resonator coupler for subcutaneous implants has been developed with a new impedance matching pattern added to the conventional loop antenna. The tuning element of a concentric metal pad contributes distributed capacitance and inductance to the planar inductive loop and improves resonance significantly. It provides a better qualify factor for resonant coupling and a much lower reflection coefficient for the implant electronics. Practical constraints are taken into account for designs including the requirement of operation within a regulated frequency band and the limited thickness for a monolithic implant. In this work, two designs targeting to operate in the two industrial, scientific, and medical (ISM) bands at 903 MHz and 2.45 GHz are considered. The tuning metal pad improves their resonances significantly, compared to the conventional loop designs. Since it is difficult to tune the implant antenna after implantation, the effects of tissue depth variations due to the individual’s surgery and the appropriate implant depths are investigated. Simulations conducted with the dielectric properties of human skin documented in the literature are compared to measurements done with hydrated ground pork as phantoms. Experiments and simulations are conducted to explain the discrepancies in frequency shifts due to the uses of pork phantoms. The design method is thus validated for uses on human skin. A noninvasive localization method to identify the implant under the skin has been examined and demonstrated by both simulations and measurements. It can efficiently locate the subcutaneous implant based on the high quality-factor resonance owing to the tuning elements in both implant and transmitter couplers. The planar resonant coupler for wireless power transfer shows good performance and promise in subcutaneous applications for implants.

## 1. Introduction

Modern electronic implants have advanced functions and reduced sizes significantly by the integration of low-power electronics. Targeting better management of chronic diseases, sensing, recording, and electrical stimulation have been considered to be incorporated into a single device, which will require signal and data communication, remote control, and battery charging capabilities. Furthermore, battery size and capacity can be greatly reduced by efficient wireless charging, or the battery can be completely eliminated with wireless powering. With supercapacitors instead of a battery [1,2], electric circuits and electrodes can be made on a flexible substrate and packaged with lamination. This opens a new class of implants that can be sufficiently thin and flexible to be implemented subcutaneously or interstitially for electrical and electrochemical sensing or stimulation [3,4,5,6,7].

Conventional wireless power transfer methods for implants utilize coils for inductive coupling [8,9,10,11,12,13]. This has been implemented for the charging function in several FDA-approved neurostimulators. Some have been in clinical studies. Commercially available ones have been compared on their efficacies and costs in practical uses [14,15,16,17,18,19,20,21]. Coil antennas utilizing magnetic field coupling to produce electric currents, as compared to antennas based on electric field coupling, can provide higher transfer powers. Electric field coupling antennas are constrained in designs by their dimensions in scales of the wavelengths at their operating frequencies. Magnetic coupling with a sufficiently high mutual inductance, typically achieved by large self-inductance with a high turn number of coils, can achieve good power coupling without the limitation related to wavelength. In pacemakers or neuro-stimulators, the cross-section of coils is kept limited in order to keep the implants compact so that the incision for implantation procedures can be small to prevent patients from unnecessary pain. Typical stimulators have volumes of 14–40 cm^3^ [22]. The smallest FDA-approved one with a conventional shape has a 47.2 × 57.1 mm cross-section, so the coil cross-section is then limited to it [14,23]. The coil antenna can be larger for the external handheld device, acting as a reader and power transmitter. However, its dimensions are still limited by operation convenience, as the patient has to hold it by hand against the chest or waist to charge the implant for tens of minutes or even a few hours.

Typically, wireless power charging at the resonant frequency provides better efficiency. A 3-D solenoid made of winding wires with a measured self-inductance and a tuning capacitor or capacitance bank are used to achieve resonance. The operating frequency can be tuned as desired if a sufficient range of capacitance is available. Often, the inductance is determined empirically. Data transmission can be conducted through the same coil pair because the data rate for vital sign information and control command is usually low. Such solenoid coils intrinsically are bulky due to winding wires and its 3-D architecture. The operation is limited to lower frequencies along with low quality factors, not to mention it does not allow planar or monolithic configurations of implants.

From an equivalent circuit point of view, the coupling can be modeled as a transformer. The mutual inductance and coupling coefficient vary the loading impedance to the implant coil in the near-field range for efficient energy coupling. This creates an impedance mismatch for the circuit of the entire system. This affects the transmission and reflection coefficients in the implant and transmitter sides. Although dynamic tuning can adjust the reader/transmitter for better impedance matching, it is not preferred to have automatic or manual tuning in the implant in order to avoid additional circuit complexity or size increases. This is particularly critical for planar subcutaneous or interstitial implants.

Furthermore, with the trend that implant sizes are getting smaller and future implants are moving toward planar configurations, a quick and convenient way to identify the device location inside the body is needed, especially for subcutaneous implants. The conventional way of X-ray computed tomography [24,25] takes time, adds more costs, and exposes patients to additional risks as it is by ionizing radiation. 

For subcutaneous and interstitial implants, the thickness of the device package should be as thin as possible. For example, subcutaneous electrodes are implemented for electrical stimulation of peripheral nerves to inhibit chronic pain [5]. It demonstrated substantially reduced procedural risk and improved quality of life by reducing pain without analgesia; however, the wired connection presented practicality issues for long-term uses. A permanent subcutaneous implant with wireless control and power will resolve the usability issue to optimize pain management benefits. Another example is a foldable gastrostimulator fabricated on a polyimide substrate that can be folded into a cylinder shape and inserted into a tube delivered by an endoscope into the stomach via the mouth and esophagus [3]. The device is then unfolded back to its planar shape and inserted into the stomach’s submucosal layer as a secure attachment method. The gastrostimulator delivers electrical pulses into the mucosal and submucosal layers of the stomach to modulate its motility. These devices are fabricated on biocompatible flexible polyimide substrates with planar spiral antennas for inductive coupling [7]. To keep the device size as small as possible, the coil turn numbers are limited. As an effort to increase self-inductance, the reduction of metal line width in order to increase total metal line length inevitably increases the AC resistance of metal. This reduces its quality factor and operating frequency. Furthermore, the antenna experiences variations of effective permittivity at different implant depths. Impedance matching becomes a challenge because the circuits need to offer a wide range of tuning capacitances to minimize the implant reflection coefficient at different depths in skin.

In this work, we consider practical constraints for implantation in designing the planar loop antenna for implants. The constraints include minimally-invasive surgical procedures; therefore the devices have to be as thin as possible. This addresses practical usage by patients whose implants should be small for comfort and tolerate different tissue thickness. Widely used loop antennas have been analytically studied by Storer [26] and McKinley et al. [27]. The real and imaginary parts of port impedances, equivalent resonant circuits and resonant conditions have been presented. For a single loop with a radius of *b*, the circumference *2πb* and the resonant frequencies of harmonics are closely related. However, resonant wavelengths $\lambda_{m}$ are not exactly $\lambda_{m}=2\pi b$/*m* due to impedance variations around the resonant points in which the current distributions on the metal are affected by fields, where *m* is an integer, as one would have expected. A unit-less factor *Ω* = *2ln*(*2πb*/*a*), where *a* is the half of the loop metal line width *w*, defines resonance performance at resonant frequencies in [27]. As an example, for *Ω* = *12* the resonances occur when *2πb*/*λ* = *1.069*, *2.099*, *3.123* and *4.144* for the first four resonance points, instead of the exact integers of *m* = *1*, *2*, *3*, *4*, at which the imaginary parts of loop impedances become zero or near zero while the real parts of the impedances reach local minima around the resonant frequencies [26,27]. At the resonance points, AC current establishes standing waves along the loop building up resonance. Defined in [27], at anti-resonant points between the resonant frequencies, the real parts of impedances reach local maxima when imaginary parts become zero or near zero. When this happens, little current flows in the loop and as a result magnetic fields become weaker. At higher resonant points, typically above the third resonance, the quality factors decrease significantly as the resonant and anti-resonant points become closer in frequencies.

In general, when *Ω* < *9* or *b*/*a* < *14.3*, the resonances of a single loop become insignificant. The reflection coefficient is not sufficiently low for the circuits to deliver power efficiently into the antenna. Thus, for an ideal case, *b* should be larger. However, the implants loop radius *b* is roughly pre-determined by the operating frequency, which needs to follow the industrial, scientific and medical (ISM) bands defined by the International Telecommunication Union (ITU). In our cases, they are at 903 MHz or 2.45 GHz. Therefore, *b* cannot be as large as one might wish. Increasing *Ω* by decreasing *a* makes fabrication more difficult and costly. A narrower metal line also increases AC resistance and power dissipation and thus reduces the loop quality factor. These limitations have restricted the performance of planar loop antennas in implants. 

**Figure 1:**
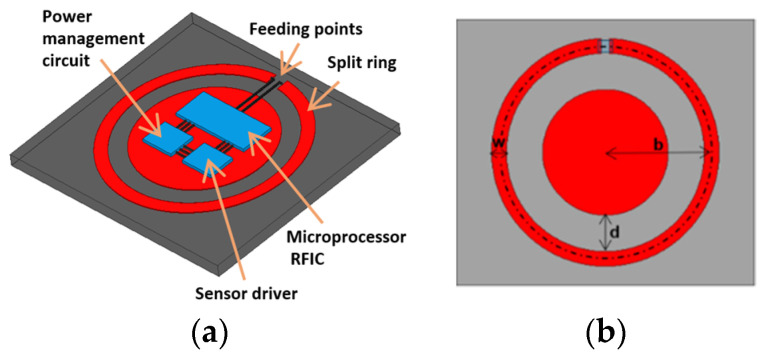
(**a**) The device architecture of the resonate coupler. The center pad reserves a space to integrate circuitry. (**b**)The coupler dimensions: the radius *b*; the loop width *w = 2a*; and the tuning gap *d* between the pad and ring.

We proposed a tuned loop antenna structure for near-field coupling applications, given the aforementioned limitations on dimensions. Preliminary results have been presented in the Wireless Power Transfer Conference and this manuscript is an extended version of the conference paper [28]. The antenna is based on a simple planar loop similar to the wire loop in [27]. The spectral shapes for impedances can be calculated by analytical forms, in order to determine resonant frequencies. The new coupler has a metal pad embedded in the center for tuning by the gap between pad and loop. The center pad also serves as the space to accommodate circuits, as shown in Figure 1a, making the implant compact. The center pad contributes distributed shunt capacitances and mutual inductances to the loop impedance. With appropriate gap distance, the resulting overall impedance matches the port at the targeted resonant frequency. In this work, we focus on demonstrating the designs for the first and second resonances at 903 MHz and 2.45 GHz, respectively. Their reflection coefficients at different implant depths under the skin are studied. The discrepancies due to the differences of dielectric properties in the documented human skins and phantoms made of ground pork are investigated. The resonant antenna can also serve as a beacon for locating the implant noninvasively by a scanner placed on the skin. The capability and resolution are examined.

## 2. Resonate Coupler Designs

The coupler consists of a loop with a split gap as the excitation port and a center metal pad, shown in Figure 1b. The center pad adds distributed capacitances, as electric fields are established across the gap between metal patterns along the loop. Near resonance points, the current flows on the loop produce magnetic fields in the gap. The same fields induce currents on the center pad but with an opposite direction. Thus, negative mutual inductance is presented to the equivalent circuit and reduces the total inductance. The spacing *d* between the loop and pad affects how much capacitance and inductance are introduced. By varying *d*, the impedance and reflection coefficient can be tuned. 

Two designs are conducted for the ISM bands of 902–928 MHz and 2.4–2.5 GHz. For the reason of comparison, we choose *Ω* = *9*, by which the loop width is not too narrow to fabricate. The devices are simulated and fabricated on single-layer FR4 substrates, which have a dielectric constant of 4.4 and a thickness of 1.5 mm. The first resonant frequency of 903 MHz is predicted for the ring with a radius of 13.15 mm and width of 1.8mm, while 2.45 GHz for the second resonance with a ring radius of 8.9 mm and a 1.16-mm width. 

## 3. Experiment and Simulation Results

**Figure 2:**
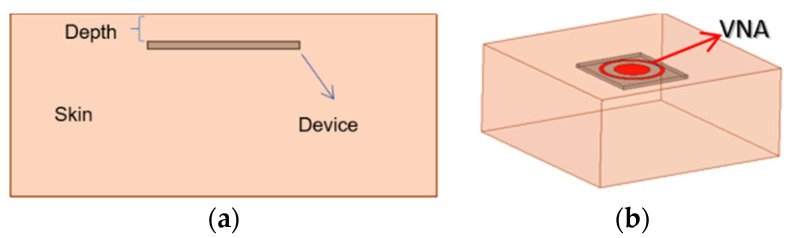
Simulation and measurement configurations. (**a**) The side view. (**b**) The 3-D view.

Human tissue permittivities are obtained from [29]. The simulation setup is shown in Figure 2. The device is inserted into the dermis layer with a variable depth, shown in Figure 2a. The antenna connects to a vector network analyzer (VNA) via a 50-Ω SMA adaptor, shown in Figure 2b. The permittivities and conductivities are frequency dependent in the finite-element simulations up to 3 GHz. The designs target an implant depth of 6 mm for both ISM bands. At 903 MHz, the documented relative permittivity and conductivity are 46.068 and 0.845 S/m, and at 2.45 GHz, 42.853 and 1.5919 S/m, respectively. 

**Figure 3:**
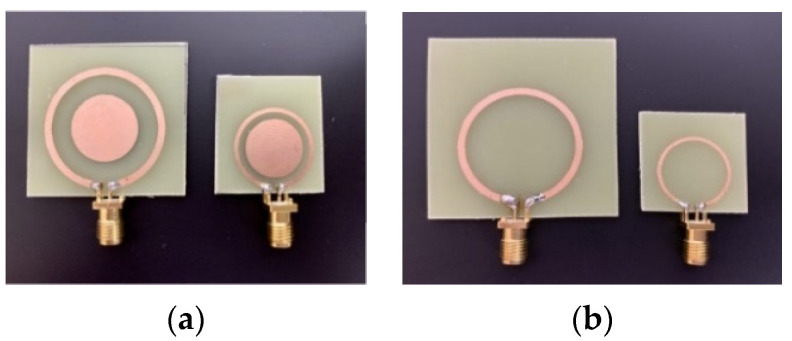
Photographs of the (**a**) tuned resonate couplers with a center pad and (**b**) single loops without the pad.

Because it is impossible to use human tissues for experiments, ground pork with about 27% of fat and 0.013 moles of saline is used as the tissue material at room temperature. The pork is packed in a cube of 100 × 100 × 50 cm^3^ as a phantom. The cube is sealed with multiple layers of plastic wrap to keep moist. Disagreements on the tissue dielectric characteristics (permittivity and conductivity) are expected. Verification for the discrepancies affected by this factor will be conducted later. Both the simulations and measurements are conducted up to 3 GHz, covering the two desired ISM bands. Photos of the devices with and without the pad are shown in Figure 3. Measurements are conducted with a PNA N5227B vector network analyzer (*Keysight*).

### 3.1. Improvement of Resonance

**Figure 4:**
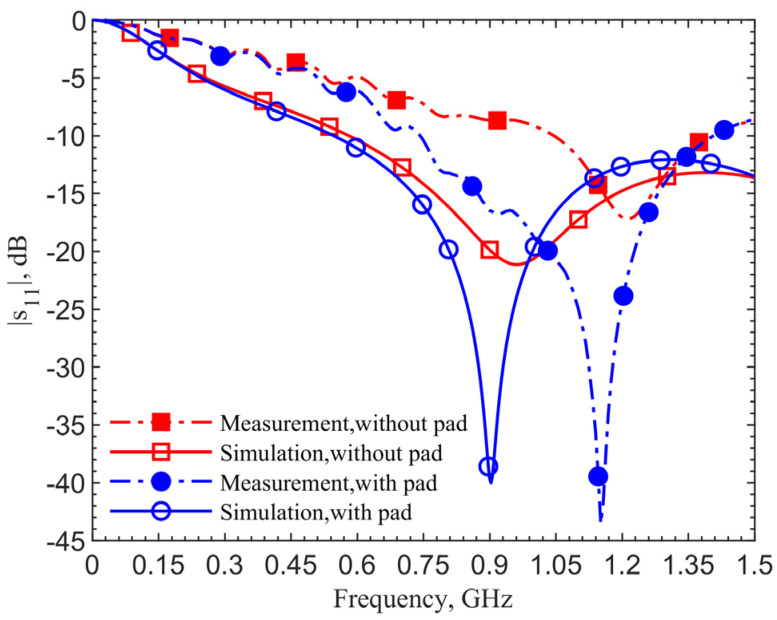
Simulation and measurement results of the resonate couplers with and without the center pad. The design is for the ISM frequency band 902–928 MHz.

Figure 4 shows the reflection coefficients for the 903-MHz couplers with and without the pad at an implant depth of 6 mm. The figure data labels (squares and circles) are used to distinguish the curves. They are not the data points. Simulations are obtained with 801 points while measurements contain 1001 points. This applies to all the comparison figures in this manuscript. 

The device with a center pad with the gap *d* = 4.5 mm, has much improved resonance performance, with the |*s*_11_| magnitude at 903 MHz in simulation improved from −21.14 to −40.03 dB, and at 1.152 GHz in measurement it improved from −17.19 to −43.54 dB, as indicated by the blue curves. From the impedance equations in [27], the loop without the pad is designed at 903 MHz; however, simulation and measurement show resonant frequencies of 960 MHz and 1.209 GHz. For the same depth of 6 mm, the resonant frequency of the loop shifts from 903 to 960 MHz, while the one with a pad does not shift in simulations. The shift shows the tuned loop has a more robust resonance so that the effective dielectric constant does not affect the resonant frequency. 

The 249-MHz frequency shift between simulations and measurements, comparing the solid lines to dashed lines, occurs due to the difference of dielectric properties between human skin in simulations and ground pork phantom in measurements. In both cases with and without the pad, the shifts are the same. This discrepancy will be verified later. Measurement results for the cases with and without the center pad clearly indicate a significant improvement in resonance. The reflection coefficients are improved from −17 to −43.5 dB, while the simulation results are improved from −24.6 to −52.9 dB. 

**Figure 5:**
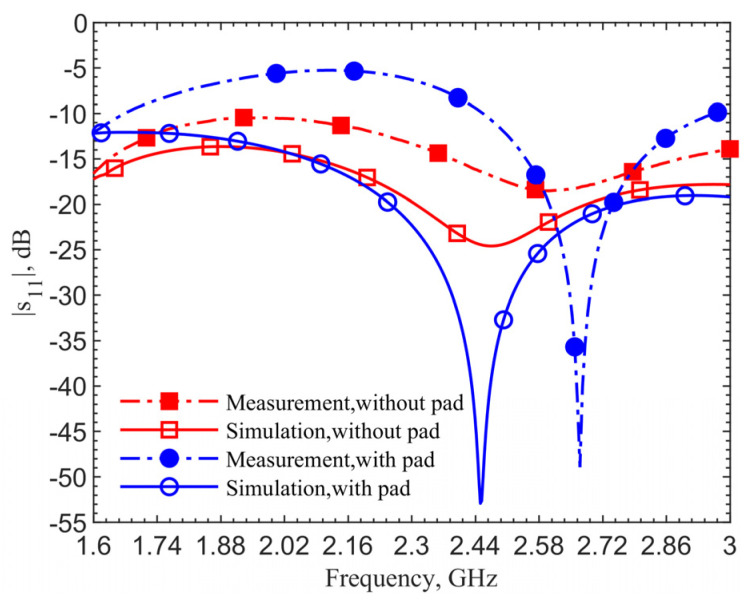
Simulation and measurement results of the coupler with and without the center pad for the 2.4–2.5 GHz designs.

Figure 5 shows simulation and measurement results for the coupler with and without the pad at the 2.4–2.5 GHz frequency band. The design targets the second resonance at 2.45 GHz. With a gap *d* = 1.5 mm, the measured reflection coefficient is improved from −18.5 to −48.9 dB. The simulation shows resonance for the case without the pad but measurement indicates a much less noticeable one. On the contrary, the cases with a pad show clearly good impedance matching; despite the pork tissue permittivity is different from that of human skin. This emphasizes the need to have a robust resonance in designs considering tissue property variations. A frequency shift of 219 MHz between simulation and measurement for the cases with the pad is also observed.

**Figure 6:**
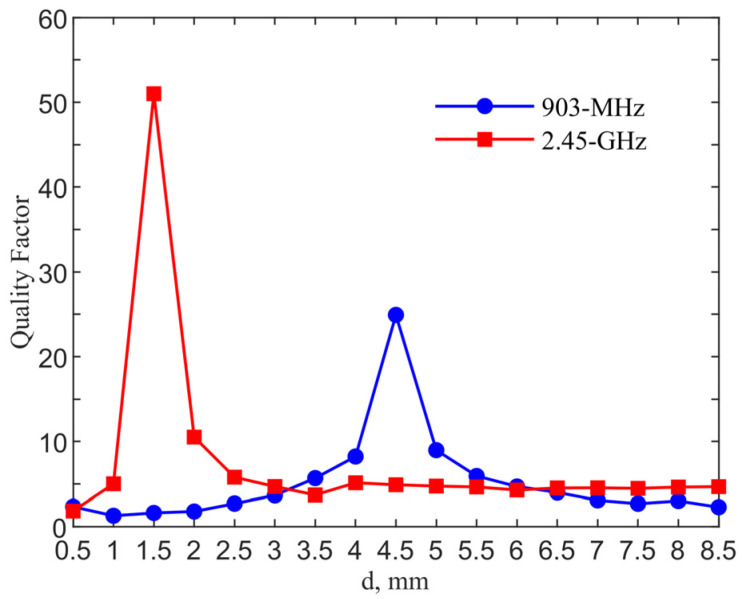
Quality factors of the loop with tuning pad at the two ISM bands for the implant depth of 6 mm.

Figure 6 shows the qualify factor comparison for the designs in two ISM bands. The quality factor is calculated from $Q=f_{0}$/Δf where $f_{0}$ is the resonant frequency and Δf is the 3-dB bandwidth. The quality factors reach maximum values of 24.91 and 51 with *d* = 4.5 and 1.5 mm for the 903-MHz and 2.45-GHz designs, as compared to those of 2.12 and 4.80 for their counter-parts of the single loop designs without a pad.

### 3.2. Performance at Different Implant Depths

**Figure 7:**
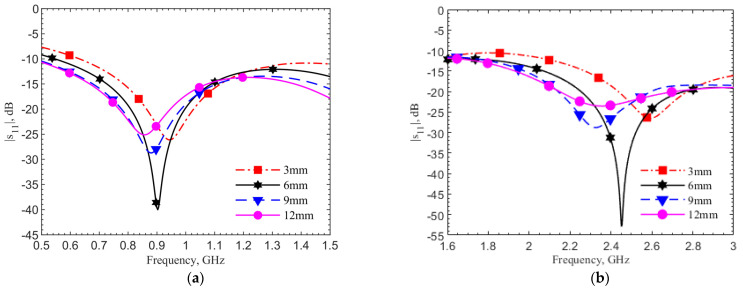
Simulations of the reflection coefficient for (**a**) the 903-MHz and (**b**) 2.45-GHz designs with tuning pad in the depths of 3, 6, 9 and 12 mm. The simulations are conducted with human skin properties, reprinted with permission from [29]. Copyright 1997 Institute of Applied Physics.

The implantation depth is designed at 6 mm for both frequency bands. In realistic scenarios, surgeons may not have a way to control the depth as precisely. Figure 7a,b show the reflection coefficient changes at depths of 3, 6, 9, and 12 mm by simulations using human skin data, respectively, for the 903-MHz and 2.45-GHz designs. In both designs, the frequency shift percentages are within 6%, while the reflection coefficients and quality factors vary. The refection coefficients for all four cases are below −20 dB. In the cases without the tuning pad, not shown for the sake of brevity, only the 6-mm cases have refection coefficients around −20 dB at the operating frequencies. This means the tuning element provides not only a better resonance at the operating frequency but also a more robust design against the depth variations.

**Figure 8:**
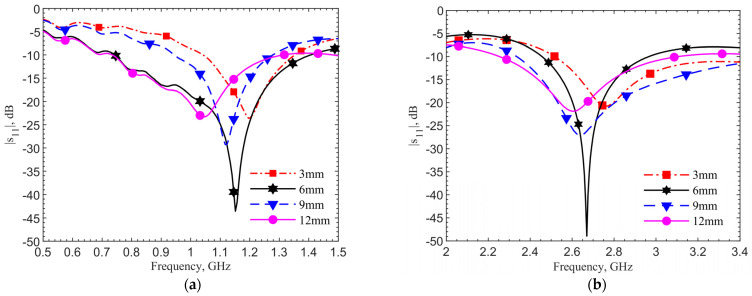
Measurements of (**a**) the 903-MHz design and (**b**) the 2.45-GHz design with tuning pad at depths of 3, 6, 9 and 12 mm in the ground pork phantom.

The devices are inserted into the ground pork phantom at different depths of 3, 6, 9 and 12 mm. The step of 3 mm is chosen because it is difficult to precisely control smaller spacing in the insertion of device into the glutinous ground pork. Figure 8 shows the measured resonances for the designs at (a) 903 MHz and (b) 2.45 GHz. They match well with simulations. Both plots show the same frequency trends at different depths. This is expected. As field distribution proportion between air and tissues changes with the implant depth, the effective permittivity experienced by the antenna affects resonance. In all four cases, the reflection coefficients are lower than −20 dB meaning good matching to the driving electronics in the implant. At the desired depth of 6 mm, the resonances are at 1.152 GHz and 2.67 GHz, respectively. The measured resonant frequencies remain to have similar frequency shifts from the respective theoretical values. This is again due to the dielectric properties between the ground pork and documented human tissues.

**Figure 9:**
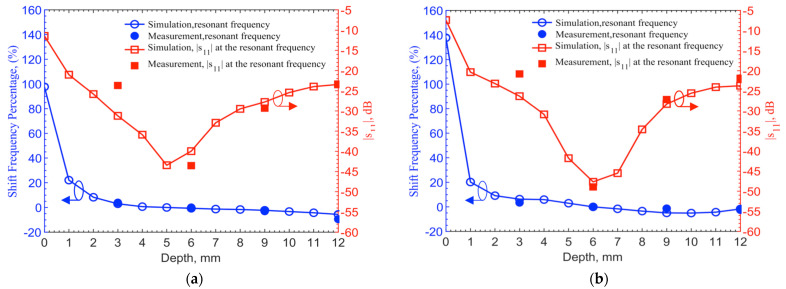
Frequency shift percentage (left y-axis) and *s*_11_ magnitude (right y-axis) in the depth of 0–12 mm for the (**a**) 903-MHz and (**b**) 2.45-GHz designs with tuning pad. Measurements in discrete depths of 3, 6, 9 and 12 mm are compared with simulation results obtained at 1-mm steps.

Simulations are conducted for depths at a 1-mm step up to 12 mm utilizing the documented frequency-dependent human skin properties [29]. 903 MHz and 2.45 GHz for the 6-mm depth are set as the reference points to find frequency shifts in different depths. For measured results, frequency shifts are extracted from the resonant frequencies of 1.152 and 2.67 GHz as the reference frequencies, which are also at the 6-mm depth. Figure 9 shows the resonant frequency shifts in percentages from their respective reference frequencies and corresponding reflection coefficients. The measured resonant frequency shifts and return losses at the four discrete points of 3, 6, 9 and 12 mm match with the theoretical results. For the depth of 3 mm, the frequency shifts match well, while the |*s*_11_| has 7 and 5 dB discrepancies for 903-MHz and 2.45-GHz, respectively. Again, this is due to the differences of permittivities and conductivities in human skin dataset and ground pork. It should also be noted that the documented human skin permittivity data was obtained by measurements on top of the epidermis layer, in which it combines all electromagnetic-wave effects from epidermis, dermis and fat layers as well as blood vessels and glands. The pork phantom is constructed with quasi-uniformly mixed fat and muscle tissues without layers. Thus, some differences between simulation and measurement are expected.

In conclusion, the implant depth should be deeper than 3 mm in order to maintain operating frequency shifts less than 5% from the designed one. To maintain a −30 dB reflection coefficient from the implant circuitry to antenna, the implant depth should be below 4 mm but above 8.5 mm. For a requirement of −20 dB, the implant depth can be relaxed to 2–12 mm. This is given that the transmitter can be tuned to reach resonance within the 5% range of the designed operating frequency.

### 3.3. Discrepancies Due to Phantom Dielectric Properties

The aforementioned discrepancies between measurements and simulations in multiple cases are observed for both designs with and without the pad, and at different depths. Similar frequency shifts of 249 and 219 MHz between theory and measurement are obtained for the 903-MHz and 2.45-GHz designs, respectively. Our hypothesis is that the permittivity and conductivity of the moist ground pork phantom to mimic human tissues are lower than those of documented human tissues [29]. Ground pork contains more fat than human skin, so the permittivities should be lower at the frequencies of interest [30,31,32]. The ground meat does not contain flows of blood or interstitial fluid, so it is less conductive [33,34]. 

**Figure 10:**
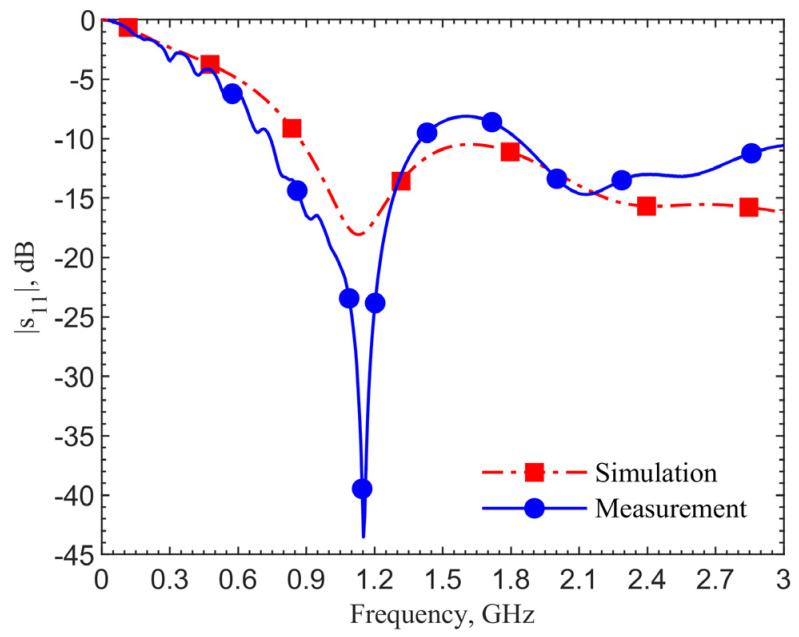
Simulations show frequency matching after a relative permittivity decrease of 25 and a conductivity decrease of 0.35 S/m.

To validate this hypothesis, we reduce the relative permittivities and conductivity of the human tissues uniformly across the frequencies up to 3 GHz and conduct iterations of simulation with the new parameters. Figure 10 shows that resonant frequencies match at 1.152 GHz, measured by using the ground pork phantom, with similar spectral shapes. In this case, the relative permittivity is decreased by 25 and the conductivity by 0.35 S/m.

**Figure 11:**
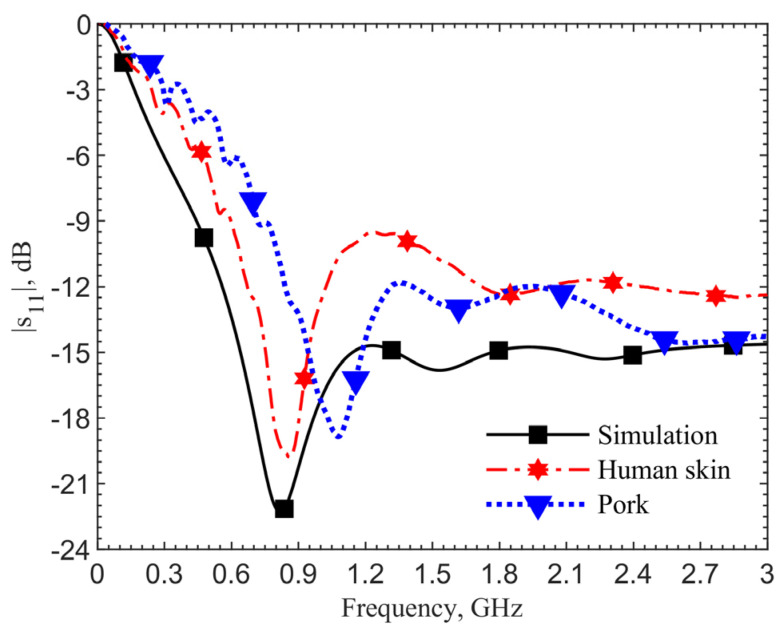
Comparison of results by the simulation utilizing the documented frequency-dependent human skin electrical properties, by the measurement on top of a person’s forearm skin, and by the measurement on the top surface of the ground pork cube.

For further validation, we compare the scenario at the zero depth. The loop directly touches the skin. Figure 11 shows the results of the simulation utilizing the human skin electrical properties (black curve), measurement with the antenna placed on the skin of a person’s forearm (red), and on the top surface of the ground pork cube (blue). The resonant frequency in the simulation is 0.82 GHz, which matches the measured 0.86 GHz on the arm’s skin. The resonant frequency is 1.08 GHz for the ground pork phantom. The frequency difference of 220 MHz between the human skin and pork also agrees with those in other depths. From these two validation results, the designs utilizing the human skin properties could work well in realistic scenarios.

## 4. Noninvasive Localization of Implant

For subcutaneous or interstitial implants, the devices become invisible after the incision heals. With advances of biocompatible materials and technologies, they will become thinner, smaller, and can be delivered by an injector that leaves a minimal scar [35,36,37], making it challenging to locate the implant sites without using sophisticated instruments such as X-ray computed tomography [38]. It is particularly important if the patients who have such implants are unconscious or in an emergency room setting. Owing to the resonance between the implant antenna and transmitter, it can be utilized in a continuous or discrete raster scan method to identify the implant.

**Figure 12:**
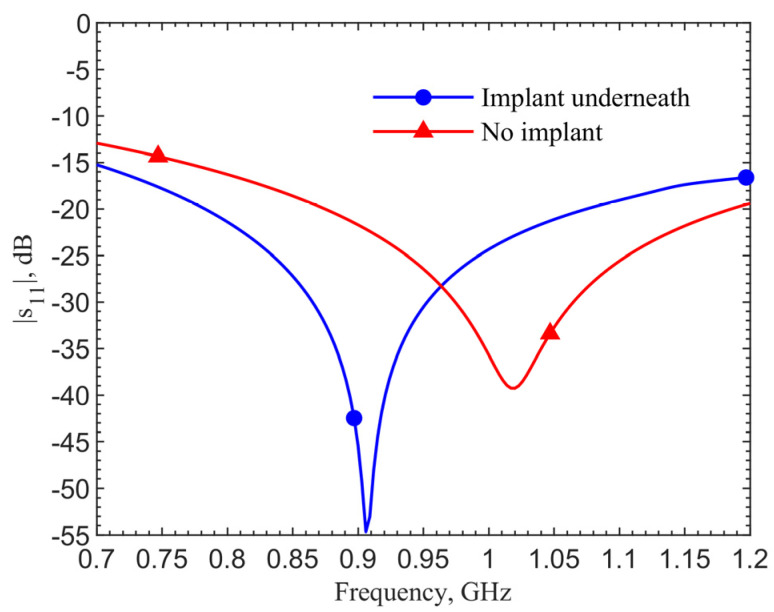
Comparison of *s*_11_ magnitude between the cases with and without implant underneath the skin.

The implant antenna is tuned to resonance at the 6-mm depth with a reflection coefficient of −58.85 dB at 906 MHz. The transmitter is then tuned to work with this implant. Shown in Figure 12, its reflection coefficient at 906 MHz is −54.65 dB. When there is no implant underneath, the resonant frequency at the transmitter becomes 1.02 GHz with a reflection coefficient of −39.27 dB.

### 4.1. Simulation

A discrete raster scan is conducted to validate the noninvasive localization concept. The implant is at a depth of 6 mm while the transmitter is placed on the skin. A 2-D map of the resonant frequencies can indicate the implant location. When the two tuned loop antennas align center-to-center, it is set as the origin point (x, y) = (0, 0). Each pixel is 10 × 10 mm^2^. The total scanning area is 110 × 110 mm^2^ with a resolution of 11 × 11 pixels.

**Figure 13:**
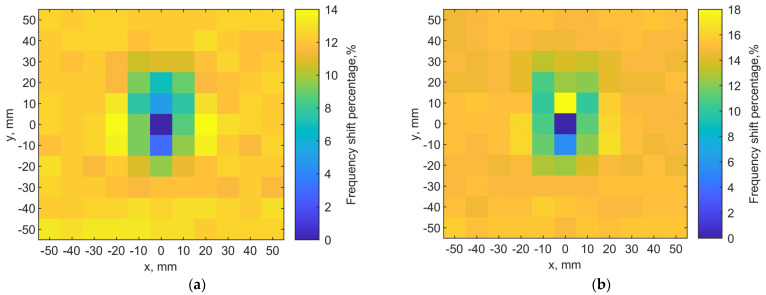
(**a**) Localization result from simulations for the implant at a depth = 6 mm. The implant is located in (x = 0, y = 0). The dark blue color pixel indicates the implant location. (**b**) Localization map with the transmitter loop port orientation rotated by 90° from the case in (**a**).

Figure 13a shows the scanning results. The color scales show the resonant frequency shifts from the designed one. It clearly shows the location of the implant antenna at (0, 0), where the resonant frequency of the transmitter is 906 MHz. Adjacent pixels (0, 10) (0, −10) have 960 and 940 MHz on the y-axis, respectively. At the edges of the map, its resonant frequencies are around 1.02 GHz.

For the fundamental mode of resonance, the magnitudes of current distribution on the loop have maximums at the port and in the middle of the loop length, while minimums are located at the first and third quarters of the loop circumference. This creates an asymmetry in radiating field distributions. Even when the two loops align center-to-center, if the port orientations are different, the field coupling becomes different, and it effectively changes the resonance. 

Because the port orientation of the implant loop is unknown after implantation, we examine two raster scanning maps. If we draw a line from the port to the center of the loop for both antennas to indicate their port orientations, the first map is created by placing the transmitter loop with the port orientations aligned with each other, as the two lines are in parallel. The result is shown in Figure 13a. The second one is obtained with the port rotated by 90°, as the two lines are orthogonal, after the first raster scan. The result is shown in Figure 13b. It is clear that the implant location can still be identified as the darkest color in the center of both maps. The asymmetric field distributions by the loops have an impact on the resonances as the surrounding pixels indicate different patterns of resonant frequencies. 

**Figure 14:**
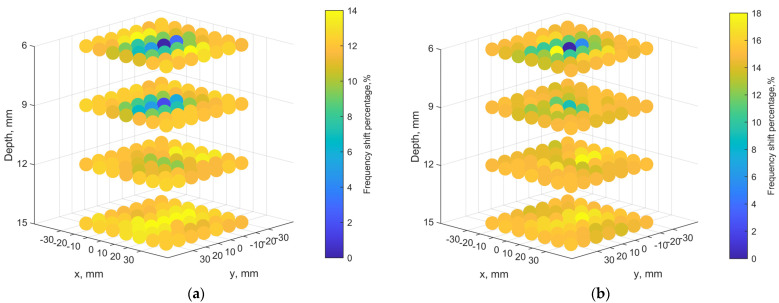
Localization maps with the transmitter loop in different depths of 6, 9, 12, and 15 mm: (**a**) at the orientation where the ports are aligned, and (**b**) at a rotated orientation of port by 90° from the cases in (**a**).

The subcutaneous implant is typically located in the hypodermics layer, and the depth is within 6 mm. In practical scenarios, the implant can be deeper. The results of mapping for an implant depth of 6, 9, 12 and 15 mm are shown in Figure 14a. Figure 14b compares the cases for orthogonal port orientation. The scanning image becomes noisy and less distinguished at 12 and 15 mm, compared to the 6- and 9-mm cases. It is obvious that when the port orientations are the same, the coupling is the strongest so the pixel differences are more distinct, comparing the same depths in Figure 14a,b. When the misalignment becomes greater, in addition to resonant frequencies becoming closer to the one without implant underneath, the frequencies fluctuate across pixels. Again, this is due to the 3-D field distributions of the tuned loop antennas in the tissues. As the implant is deeper, its fields spread out to more pixels, and the transmitter can still have some coupling effects on the implant. 

### 4.2. Measurements 

**Figure 15:**
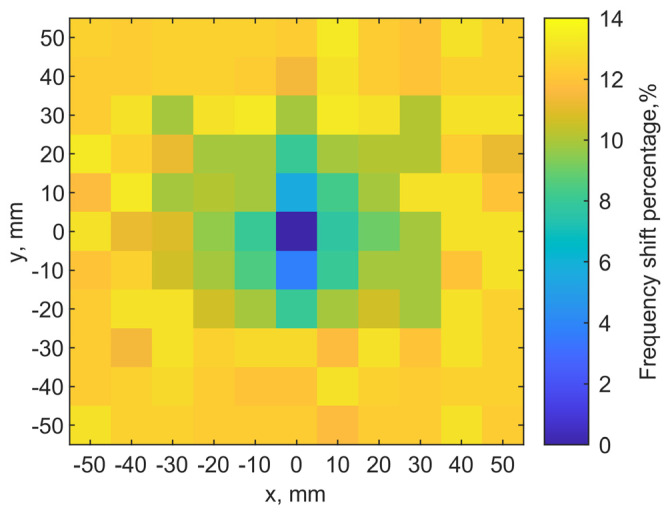
Localization result from measurements with the implant in a depth of 6 mm. The implant is located at (0, 0).

The same ground pork phantom packed in a cube of 160 mm × 160 mm × 100 mm is used for experiments at room temperature. The phantom dimensions are the same as those in simulations. S-parameters are extracted at 1001 sampling points from 0 to 3 GHz to identify resonance. The implant antenna is inserted at (x = 0, y = 0) with a depth of 6 mm. The transmitter scans the phantom on the top with a step of 10 mm. Both loops have the same port orientation. Figure 15 shows the measured map similar to Figure 13a. It indicated the location of the implant clearly (dark blue pixel). Thus, it is validated that the raster scan for resonance can be used to locate the implant. 

**Figure 16:**
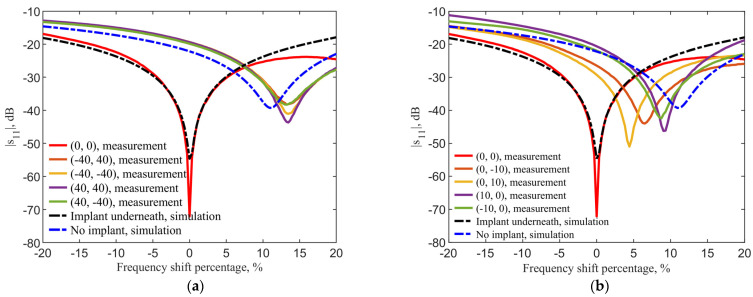
Comparison of normalized frequency shifts between measurements and simulations (**a**) at the center and near corners; and (**b**) at the center and four adjacent pixels. The pixel location is indicated as (x, y) on the map.

Figure 16a compares the spectral shapes measured at the center and at four corners. Due to the difference of dielectric properties between the documented human tissues and ground pork, the resonant frequencies shift by the same amount of 249 MHz between simulations and measurements. The frequencies are normalized for the measured (solid lines) reflection coefficients in Figure 16a to compare with the theory (dashed lines). The normalized frequency shifts show a 13.22% shift between the case with the implant underneath and that without implant. The simulations show a shift of 11.8%. The discrepancy of 1.42% is likely contributed by the differences in dielectric properties of ground pork phantom and human skin dataset. 

While aligned, the measured reflection coefficients |$s_{11}|$ in the implant and transmitter are −30.1 dB and −72.1 dB, as compared to the simulation results of −31.25 dB and −54.65 dB, respectively, while the transmission coefficient |$s_{21}|$ with implant underneath is −13.45 dB in measurement and −13 dB in simulation. When there is no transmitter above the implant, the measured reflection coefficients |$s_{11}|$ in the implant becomes −53.1 dB, as compared to the simulation result of −58.85 dB. 

Figure 16b compares the center and four adjacent pixels. Compared to the perfect alignment between implant and transmitter, the adjacent four pixels have resonance frequency shifts between 4.43% and 9.3% in the locations (0, 10) and (10, 0), respectively. The measured reflection coefficients |$s_{11}|$ in the transmitter becomes −50.9, −44.0, −42.4, −46.2 dB instead of −72.1 dB. The obvious frequency shift patterns from Figure 16a are due to the stronger field coupling when the transmitter and implant are closer, and the asymmetric field distributions of the transmitter in tissue. Nonetheless, the tuned transmitter can sensitively provide a distinguished frequency shift between the cases with or without an implant underneath despite the similar field distributions among adjacent points. 

The frequency shifts around the implant location are distinct enough with the 1-MHz resolution with 1001 sampling points from 0 to 3 GHz. The magnitude of reflection coefficients in the adjacent pixels can also provide further clarification for the implant location. Given a finer resolution in frequency in a narrower frequency sweep range, say between −5% and 20%, to measure resonance, the localization resolution can of course be increased. 

**Figure 17:**
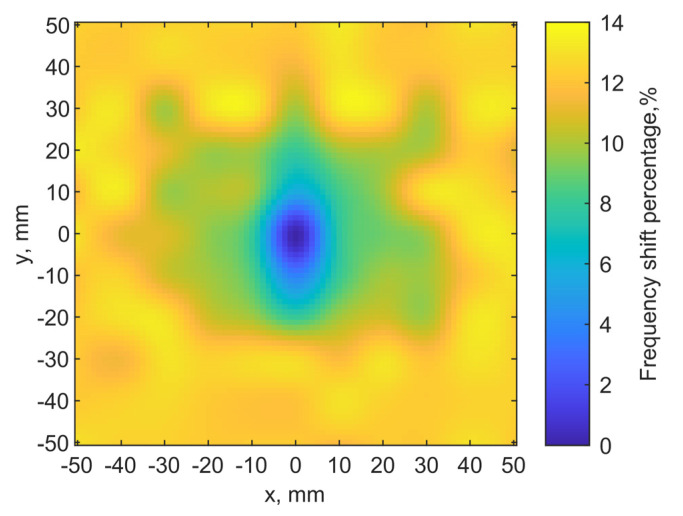
Rendering results using the cubic spline interpolation from measurement data at depths of 6 mm.

Without increasing spatial or spectral resolutions, cubic spline interpolation is used to highlight the implant location pixels for better visualization and identification [39]. As shown in Figure 17a, the implant location can be more easily identified, compared with Figure 15. Shape matching technique [40] along with the cubic spline interpolation can probably further enhance recognition of location. 

## 5. Discussion and Conclusions

In this paper, we introduced a tuning element for a planar loop-shaped split ring coupler. The center pad with an adjustable gap to the loop can tune the distributed reactance for better impedance matching. This improves the resonance performance significantly making it more suitable for wireless power and data transmission. The planar device architecture with the metal pad in the center also serves for electronics integration. The loop can feed directly into the power management circuit, which connects to a microprocessor and sensor integrated circuits. Two designs for ISM bands 903 MHz and 2.45 GHz with a targeted implant depth of 6 mm are demonstrated. Both simulations and corresponding measurements verified the concept, as resonance is significantly improved while satisfying practical constraints for subcutaneous implants. The much improved reflection coefficient of the implant also makes it more energy efficient. 

The discrepancies between simulations, conducted by documented human tissues parameters, and experiments conducted by moist ground pork phantoms, are examined and explained. The experiments validate the method, allowing us to explore the effects of different implantation depths. 

Because implant depth can vary in practical scenarios, the effects on resonant frequency and reflection coefficient in different depths up to 12 mm have been examined. An implant depth between 4 and 8.5 mm can maintain its resonant frequency within less than a 5% shift from the designed operating frequency, and satisfy the requirement of reflection coefficient for implant circuitry lower than −30 dB. Such implantation tolerance provides stable performance in practical cases.

A noninvasive method to locate the implant utilizing resonance between two coils is also demonstrated. Owing to the better resonance in the implant and transmitter loops by their tuning center pads, raster scanning by the transmitter can identify the implant location with the resonant frequency. Both finite-element simulation and measurement results verify the localization concept. The relative orientations of the loops at various depths are also investigated, showing robust performance. The concept of using a transmitter/reader loop to identify subcutaneous implants can be cost effective and less risky, and quick compared to conventional X-ray methods. 

## Data Availability

No public data disclosure.
